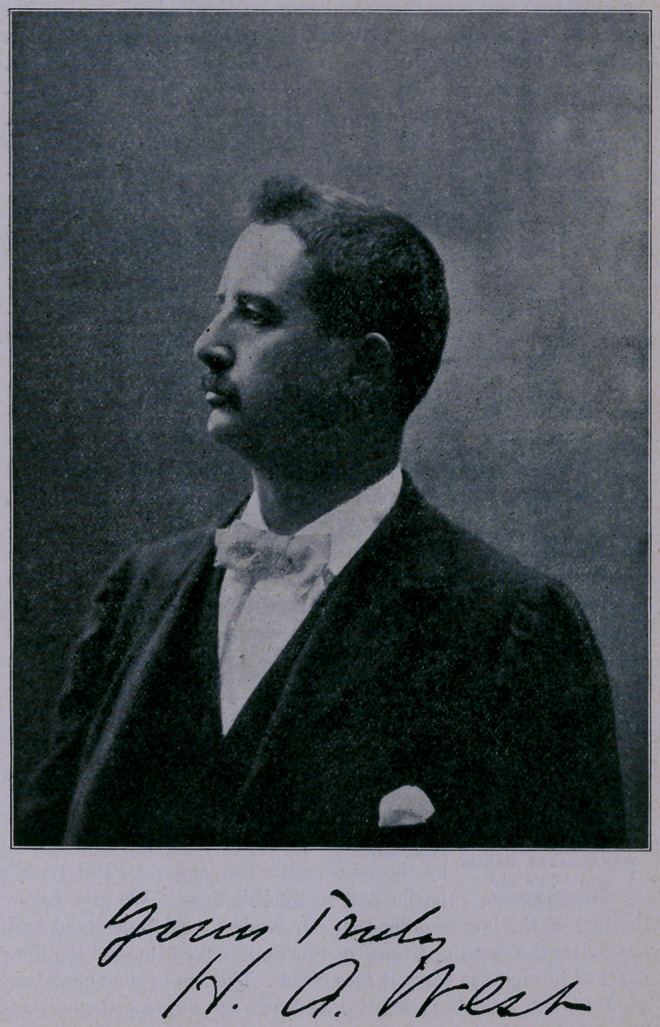# Death of Dr. H. A. West

**Published:** 1904-01

**Authors:** 


### TEXAS MEDICAL JOURNAL.

AUSTIN, TEXAS.

A MONTHLY JOURNAL OF MEDICINE AND SURGERY.

EDITED AND PUBLISHED BY
F. E. DANIEL, M. D.
Mrs. F. E. DANIEL, Managing Editor.

ASSOCIATE EDITOR:
WITTEN BOOTH RUSS, M. D., San Antonio, Texas.

Published Monthly at Austin, Texas. Subscription price $1.00 a year in advance
Eastern Representative: John Guy Monihan, St. Paul Building, 220 Broadway, New York City.
Official organ of the West Texas Medical Association, the Houston District Medical Association, the Austin District Medical Society, the Brazos Valley Medical Association, the Galveston County Medical Society, and several others.

### DEATH OF DR. H. A. WEST.

Dr. Hamilton Atchison West, of Galveston, died in New York, at the residence of his brother, Dr. Jas. N. West, at 7:40 p. m., December 30th (ult.). His hosts of attached and admiring friends throughout the South, and especially in Texas, were shocked and grieved on receipt of the intelligence of his death. Dr. West was greatly loved and admired by those who knew him well, and only those whom he admitted to intimate personal acquaintance understood him and appreciated him; for, to strangers and to mere acquaintances he was somewhat peculiar in a certain blunt, but never discourteous, manner. To his friends he was geniality itself. He was a delightful companion, a very interesting personality. He was a splendidly informed and well equipped all around practitioner and a general scholar such as is not found every day in the ranks of medicine. He enjoyed life, loved his friends and his family, and at the annual gathering of the profession in convention of the State Medical Association of Texas he was a central and conspicuous figure. He entered into the discussion of scientific papers read at those meetings with a zest and intelligence which always insured him attentive audience. Socially, at the annual banquets, he enjoyed himself, and often shone to advantage in the wit and repartee that attended the popping of champagne. We will surely miss him. It is well known that he was, and for ten or more years had been, the Secretary of the State Association. He died of Bright's disease. It looks like the "irony of fate," as he had made a specialty of the treatment of that malady.

Yours Truly
H. A. West

Dr. West was born in Kentucky, near Lexington, in 1853, and was just fifty years old; in the very prime of life. He was a graduate of the Louisville Medical College, class of 1874-75. Settling in Galveston in 1875, he soon became a popular and successful practitioner; and upon the organization of the Medical Department of the University of Texas, in 1890, he was chosen by the Regents to fill the important chair of Theory and Practice of Medicine. He was a popular teacher, much beloved by his classes. This position he resigned in 1899, having filled the chair about nine years, to give his attention to his private practice and the arduous duties of Secretary of the State Medical Association. The successful reorganization of the profession of Texas in accordance with the plan of the American Medical Association, a labor demanding much time and study and energy, was more the work of this indefatigable man than of any score of others, and had he done nothing else for them he should, and will, live in their hearts and their memories. Dr. West contributed many valuable papers to the Association's volume of Transactions.

In 1878 he was married to Miss Sarah Davenport. By her he had four children, all of whom survive her and him. Mrs. West died in 1893, and in 1895 Dr. West was married to Mrs. Mary Ella Tuller, of Galveston. By this marriage one child was born, and is now living, Mary Buckley West. Mrs. West survives the doctor. Maj. Barry West, of the U. S. Army; Dr. J. N. West, of New York; Charles West, of Corsicana, Texas, and Joseph West, of Washington, are his brothers; and Mrs. Marshall, of Kentucky; Mrs. Lee Sellers, of New York; Mrs. Dannota, of Oklahoma, and Miss Georgie West, of New York, are his sisters. Dr. West's remains were interred at Galveston. Peace to his ashes.

## Figures and Tables

**Figure f1:**